# Propose a Wall Shear Stress Divergence to Estimate the Risks of Intracranial Aneurysm Rupture

**DOI:** 10.1155/2013/508131

**Published:** 2013-09-26

**Authors:** Y. Zhang, H. Takao, Y. Murayama, Y. Qian

**Affiliations:** ^1^Australian School of Advanced Medicine, Macquarie University, Sydney, NSW 2109, Australia; ^2^Jikei University School of Medicine, Tokyo 105-8461, Japan

## Abstract

Although wall shear stress (WSS) has long been considered a critical indicator of intracranial aneurysm rupture, there is still no definite conclusion as to whether a high or a low WSS results in aneurysm rupture. The reason may be that the effect of WSS direction has not been fully considered. The objectives of this study are to investigate the magnitude of WSS (|WSS|) and its divergence on the aneurysm surface and to test the significance of both in relation to the aneurysm rupture. Patient-specific computational fluid dynamics (CFD) was used to compute WSS and wall shear stress divergence (WSSD) on the aneurysm surface for nineteen patients. Our results revealed that if high |WSS| is stretching aneurysm luminal surface, and the stretching region is concentrated, the aneurysm is under a high risk of rupture. It seems that, by considering both direction and magnitude of WSS, WSSD may be a better indicator for the risk estimation of aneurysm rupture (154).

## 1. Introduction

Rupture of intracranial aneurysm (IA) is a widely studied topic. It is reported that about 5% of adults have unruptured IAs [1–3]. Though the rupture rate of IAs is not high [4], it causes serious consequences including disability and mortality [5]. On the other hand, current treatments of IAs also carry a risk. Thus, accurate assessment of IA rupture is essential for clinicians to balance the risk of surgery against the risk of natural IAs rupture [6, 7].

Research into risk factor for IAs rupture has been reported for many years, including the effect of aneurysm size, geometry, location, and others [8, 9]. Large-sized aneurysms were considered to be under high risk of rupture. However, recent studies have shown that many ruptured aneurysms were small in size [9, 10]. In order to improve estimations of risk based on medical images, multiple geometric factors were proposed, such as aspect ratio (AR), size ratio (SR), and other factors. The statistic significance of these geometric factors to aneurysm rupture has been discussed in previous publications [11–13]. In addition to performing image diagnosis, computational fluids dynamics (CFD) technology is available to analyse hemodynamic characteristics inside the artery and aneurysm [14]. The magnitude of wall shear stress (|WSS|) has been proposed as a quantitative indicator for the flow characterization of ruptured cerebral aneurysms. Cebral et al. [15] performed CFD for a total of 62 cerebral aneurysms at various locations. They found that high-speed narrow jet flows were commonly observed in ruptured cases, which caused high |WSS| at the inlet area of the aneurysm [15]. Shojima et al. studied twenty middle carotid artery (MCA) aneurysms. Usually, spatial averaged |WSS| was higher within ruptured aneurysms than that in the parent artery. However, they also indicated that |WSS| was markedly reduced at the top of or within a bleb area of the ruptured aneurysm [13, 16]. Thus, it is not clear whether high or low |WSS| induces aneurysm rupture; some argue that high |WSS| induces aneurysm rupture [17], while others claim that low |WSS| at aneurysm dome is dangerous [18].

It may be important to note that wall shear stress (WSS) is a vector. Therefore, WSS must not be considered in terms of not only magnitude but directionality as well. In this paper, authors propose a new concept of wall shear stress divergence (WSSD), which takes into account both the gradient and direction of WSS and can be used to identify “tensile” and “compressive” regions on the aneurysm surface. Two risk factors based on WSSD distribution are also derived in order to analyse the characteristics of ruptured aneurysms.

## 2. Materials and Method

### 2.1. Patients

Nineteen patients with middle sized ICA aneurysms were observed continuously by a three-dimensional computer tomograph angiograph (3D-CTA). For ruptured-IAs, the images were obtained during the observation of 5 months on average before the occurrence of rupture, generally at the time of the patient's last clinical visit. The three patients who had subsequent aneurysm rupture (age range, 62–71 years; aneurysm size range, 5.3–7.7 mm) are cases 1–3 in this study. The other 16 patients were stable at followup. Their age range was between 40 and 78 years, with aneurysm being between 3.0 and 9.0 mm in size.

### 2.2. Wall Shear Stress Divergence

Wall shear stress divergence is expressed as following equation

(1) $$\begin{array}{c} \text{WSSD}=\mathrm{div}\left(\overset{\to }{\text{WSS}}\right)=\frac{\partial {\text{WSS}}_{i}}{\partial x_{i}},\;i=1,2,3, \end{array}$$

where WSS_*i*_ are wall shear stress components in *i* directions. If WSSD has a positive value, the net effect of WSS is to stretch the aneurysm surface; otherwise, WSS is compressing the aneurysm surface. The magnitude of WSSD represents the intensity of stretching or compressing.

### 2.3. WSSD Based Risk Factors

#### 2.3.1. Wave Centre of Positive WSSD (WSSD^+^), Negative WSSD (WSSD^−^), and *|*WSS*|*

**Figure 1:**
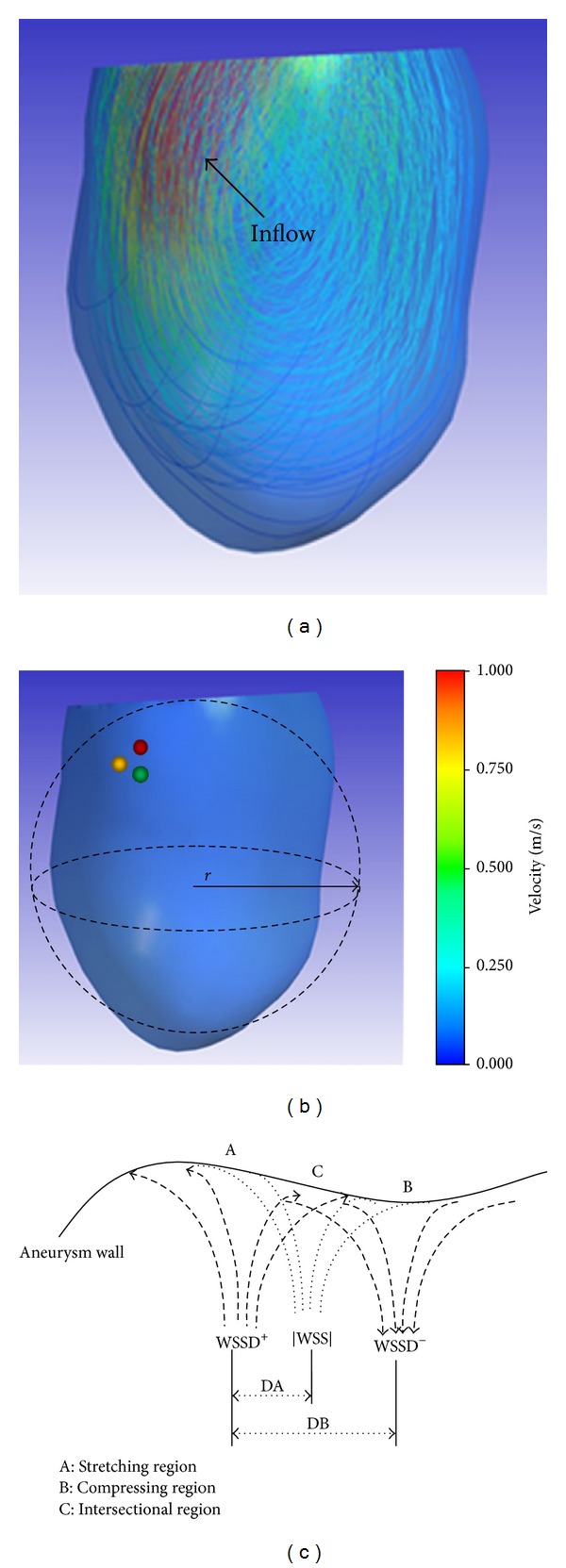
Inflow and stress centres. (a) Flow pattern. (b) Wave centres of WSSD and |WSS|; red ball: the coordinate center of WSSD^+^, green ball: the coordinate center of WSSD^−^, and yellow ball: the coordinate center of |WSS|. (c) Different actions of WSSD^+^ and WSSD^−^.

As the blood flow slows down after entering the aneurysm (shown in Figure 1(a)), all stresses decay to the surroundings in the form of a wave attenuation.  Figure 1(b) shows the wave centre of WSSD^+^ (red ball), WSSD^−^ (green ball), and |WSS| (yellow ball), respectively. WSSD^+^ and WSSD^−^ can be treated as two waves propagating in different directions. The former is stretching the aneurysm surface; the latter is compressing the aneurysm surface. In one cardiac cycle, the net effects of WSSD on aneurysm volume are counteracting (Figure 1(c)).

If the wave centers of |WSS| and WSSD^+^ are at close locations, which is dangerous, because the combination of WSS and its gradient will result in a potentially dangerous remodelling of the aneurysm [19]. On the other hand, if the wave center for both WSSD^−^ and WSSD^+^ are close to one another, the two components will compete at their boundaries (C region in Figure 1(c)), resulting in a constant shift in character for the WSS at the boundary, from “stretching” to “compressive”. This may cause a shift in flow reversal which may be adverse to the survival of endothelial cells [20]. Thus, DA and DB and the centre distance of WSSD^+^ to |WSS| and WSSD^−^ are introduced to estimate the risk of aneurysm rupture (Figure 1(c)).

#### 2.3.2. Risk Factor A

Risk factor A (RFA) is given by the following equation:

(2) $$\begin{array}{c} \text{RFA}=\frac{r_{\text{effect}}}{\text{DA}}\frac{r_{\text{effect}}}{\sqrt{{\left(x_{|\text{WSS}|,i}-x_{{\text{WSSD}}^{+},i}\right)}^{2}}},\;i=1,2,3, \end{array}$$

where *r*_effect_ is the effective radius of the aneurysm (Figure 1(b)), which is given by the following equation:

(3) $$\begin{array}{c} r_{\text{effect}}=\sqrt[{3}]{\frac{3V}{4\pi }}. \end{array}$$

*V* is the volume of aneurysm. *x*_|WSS|,*i*_ and *x*_WSSD^+^,*i*_ are the centre of |WSS| and WSSD^+^, respectively, given by the following equations:

(4) $$\begin{array}{c} x_{|\text{WSS}|,i}=\frac{1}{T}\int_{0}^{T}\frac{\sum_{j=1}^{N}x_{i}\times {\left|\text{WSS}\right|}_{j}}{\sum_{j=1}^{N}{\left|\text{WSS}\right|}_{j}}dt,\;i=1,2,3, \end{array}$$

(5) $$\begin{array}{c} x_{{\text{WSSD}}^{+},i}=\frac{1}{T}\int_{0}^{T}\frac{\sum_{j=1}^{N}x_{i}\times {{\text{WSSD}}^{+},}_{j}}{\sum_{j=1}^{N}{{\text{WSSD}}^{+},}_{j}}dt,\;i=1,2,3, \end{array}$$

where *T* is the time period of cardiac cyclic, *j* is the indicator of mesh point, and *N* is the total mesh number.

#### 2.3.3. Risk Factor B

Risk factor B (RFB) is given by the following equation:

(6) $$\begin{array}{c} \text{RFB}=\frac{r_{\text{effect}}}{\text{DB}}\frac{r_{\text{effect}}}{\sqrt{{\left(x_{{\text{WSSD}}^{+},i}-x_{{\text{WSSD}}^{-},i}\right)}^{2}}},\;i=1,2,3, \end{array}$$

where *x*_WSSD^−^,*i*_ is the wave center of WSSD^−^. Similar to (5), *x*_WSSD^−^,*i*_ is given by the following equation:

(7) $$\begin{array}{c} x_{{\text{WSSD}}^{-},i}=\frac{1}{T}\int_{0}^{T}\frac{\sum_{j=1}^{N}x_{i}\times \text{WSS}{\text{D}}^{-}{,}_{j}}{\sum_{j=1}^{N}\text{WSS}{\text{D}}^{-}{,}_{j}}dt,\;i=1,2,3. \end{array}$$

### 2.4. CFD Modeling

The conservation equations for 3D unsteady laminar flow with rigid wall boundary conditions were solved using an open source CFD code (OpenFOAM, http://www.openfoam.com/). The general form of the unsteady state equation is represented as

(8) ∂∂t(ρui)+∂∂xj(ρuiuj)  =−∂p∂xi+∂∂xj[μ(∂ui∂xj+∂uj∂xi)]+Si,∂ρ∂t+∂∂xj(ρuj)=0,

where *ρ* represents the density, *P* is the static pressure, *u*_*i*,*j*_ are velocity components, and *μ* is the dynamic viscosity. Being a second-order derivative of velocity, WSSD needs a highly accurate scheme to compute. In this study, a fourth-order difference scheme was used to discrete the diffusion term in (8) (OpenFOAM, http://www.openfoam.com/). Blood was assumed to be a Newtonian fluid with density of 1050 [kg/m^3^] and dynamic viscosity of 0.0036 [Pa · s].

In order to reduce the entrance/exit effect of CFD, the inlet and outlet of the calculation domain were extended distally in the normal downstream direction to about 10 cm. At the inlet boundary, considering that the ICA of those patients were of similar size, uniform velocity calculated from the average of ICA flow ratios (measured to be approximately 125 mL/min) [21] was introduced as the boundary condition. A zero pressure condition was used at the outlets. In the post process, nondimensional analysis was conducted to further minimize the numerical uncertainties. Grid independent study was performed in our previous publications [22, 23]. In order to accurately measure WSS at near-wall region, the body-fitted prism layers were generated near the vessel walls to improve the resolution of the relevant scales in fluid motion. There were five layers generated with an average nodal space, increasing by a ratio of 1.2. The distance from the first layer to the vessel surface was fixed at 0.02 mm.

## 3. Results

**Figure 2:**
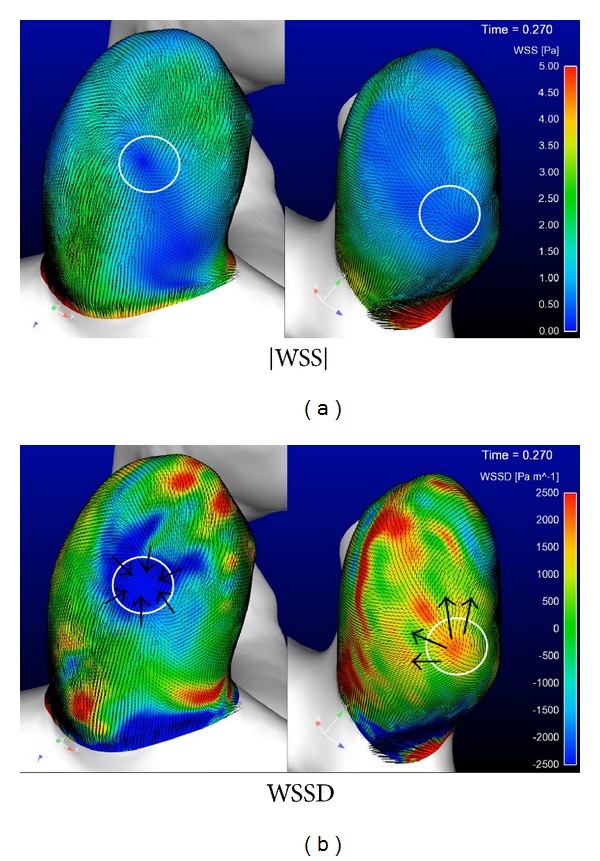
|WSS| and WSSD distributions at the systolic for a ruptured aneurysm.

For the ruptured case 1, both |WSS| and WSSD at the systolic peak (*T* = 0.27 s) are shown in Figure 2. The magnitudes of |WSS| in two locations marked by a circle were close to each other (about 1.5 Pa). However, the values of WSSD of both regions were different; in the left panel where WSS was compressing the aneurysm surface, the WSSD had a negative value (about −1000 Pa/m), while, in the right panel where WSS was stretching the aneurysm surface, the WSSD had a positive value (about 1500 Pa/m). This indicates that only the magnitude of WSS cannot directly estimate the risk of aneurysm rupture; the direction of WSS must be also considered.

**Figure 3:**
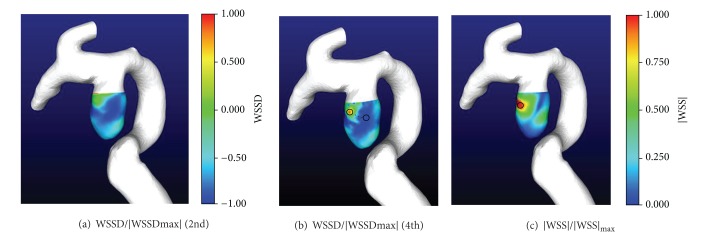
Ruptured case (case 1).

Figure 3 shows that the nondimensional distribution of WSSD and |WSS| for ruptured case 1. WSSD, calculated using a fourth-order scheme (Figure 3(b)), had a higher spatial resolution than that obtained by using second-order scheme (Figure 3(a)). The distances from the wave center of WSSD^+^ to |WSS| and WSSD^−^ were 0.4 mm and 0.6 mm, respectively. For an effective radius of aneurysm of 3 mm, (2) and (6) give the values of RFA and RFB to be 7.5 and 5.0, respectively.

**Figure 4:**
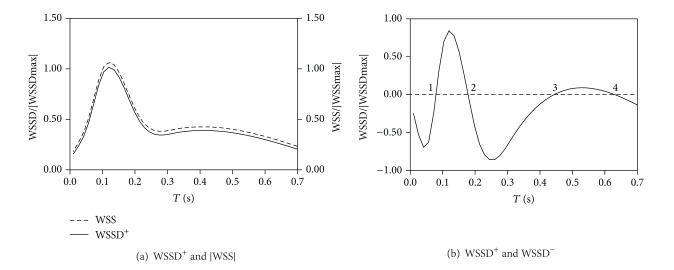
WSSD versus time for case 1.

For the same ruptured case, the variation of nondimensional |WSS| and WSSD^+^ at the nearest surface point to the |WSS| center is shown in Figure 4(a). At this point the maximum |WSS| and WSSD^+^ occur at the same time. As a result, the stretching effect of WSS reached the maximum.  Figure 4(b) shows that WSSD was observed to change four times from “stretching” to “compression” during one cardiac cycle, at the intersection region of WSSD^+^ and WSSD^−^.

**Figure 5:**
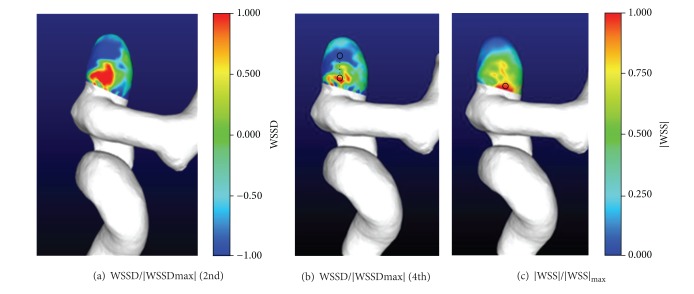
Unruptured case (case 4).

The computed results of an unruptured aneurysm (case 4) are shown in Figure 5. WSSD calculated using fourth-order scheme (Figure 5(b)) again had higher spatial resolution than that obtained by using second-order scheme (Figure 5(a)). The distance from wave centers of WSSD^+^ to WSSD^−^ reached 1.5 mm. Comparing Figures 5(b) and 5(c), the wave centres of |WSS| and WSSD^+^ are separate, with the distance in between being 1.34 mm. For the effective radius of aneurysm (4.0 mm), the calculated RFA and RFB are 2.6 and 1.7, respectively.

**Figure 6:**
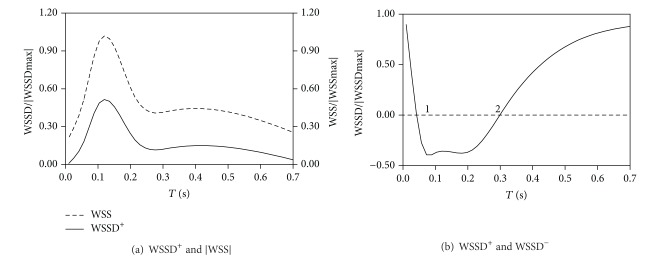
WSSD versus time for case 4.

For the same unruptured case, the nondimensional |WSS| and WSSD^+^ at the nearest surface point to the |WSS| center is shown in Figure 6(a). Though the peak of nondimensional WSS and WSSD^+^ occurs at the same time, it is seen that WSSD^+^ reaches only 50% of its peak value; that is, the stretching effect does not reach the maximum.  Figure 6(b) shows the results of WSSD at the intersectional region of WSSD^+^ and WSSD^−^. WSS only alternated 2 times between “stretching” and “compression” in one cardiac cycle.

**Figure 7:**
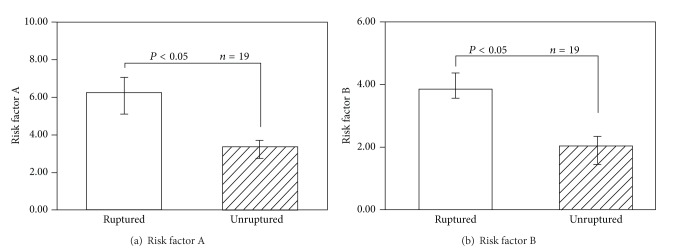
Risk factors.

The comparison of cases 1 and 4 validates our hypothesis that the value of RFA represents the “stretching” effect, and the value of RFB represents the directional change of WSS. Both RFA and RFB for ruptured case 1 are higher than those for unruptured case 4.  Figure 7(a) shows the results of averaged RFA of ruptured and unruptured groups. For ruptured aneurysms, RFA reached 6.0 ± 2.3, about 2 times that of unruptured aneurysms (3.0 ± 1.0). Similarly, the RFB of ruptured aneurysms was 3.8 ± 1.2, again about 2 times that of unruptured aneurysms (2.0 ± 1.7) (Figure 7(b)). 

## 4. Discussion

### 4.1. WSS and Aneurysm Rupture

How WSS results in aneurysm rupture has long been discussed in previous studies. It is believed that magnitude of wall shear stress |WSS| alone cannot reasonably predict the aneurysm rupture as it does not include any directional information of WSS and therefore is not able to identify whether the WSS generated is stretching or compressing the aneurysm luminal surface. Physiological studies have likewise shown that the endothelial cells lining blood vessels are subjected to WSS and responded to both magnitude and directional changes [8, 24, 25]. In any specific area, the overall effect of WSS may result in a stretching or compressive force upon the intimate surface, contributing to the different responses exhibited by the endothelial cells. This response may lead to the appearance of self-sustaining aneurysm remodelling, resulting in further aneurysm growth and rupture [26].

Oscillatory shear index (OSI) has been proposed to measure the directional changes of WSS, with high OSI correlating to aneurysm rupture [9, 27]. It should likewise be noted that OSI is only able to depict the directional change of WSS at a point and is not able to indicate whether the force generated is tensile or compressive. To address this issue, a gradient oscillatory number (GON) was developed to test the number of incidences in which the force generated varied from tensile to compressive at a certain point [28]. However, for both OSI and GON, the effect of WSS magnitude was counteracted in calculation. The combination of the high magnitude of WSS and high wall shear stress gradient (WSSG) was thus proposed to estimate the remodelling of vessel walls in response to aneurysm formation. Current reports have indicated that the intersectional area of high WSS and high gradients in WSS may represent a “dangerous” hemodynamic condition for aneurysm formation [19].

Previous research has implied that WSS magnitude alone cannot fully explain the influence of WSS on the rupture of aneurysms. WSS magnitude, gradient, and direction must all be completely and comprehensively examined, in order to reasonably estimate the risk of aneurysm formation and rupture.

### 4.2. What Are the Contributions of WSSD Based Analysis?

In the current study, we proposed a new concept of WSSD. Compared with |WSS|, WSSD considers the directions of WSS. Compared with OSI, WSSD can identify the performance of WSS at WSSD^+^ region; WSS is stretching while, at WSSD^−^ region, WSS is compressing the aneurysm luminal surface. The magnitude of WSSD represents the strength of stretching or compression.

Recent study has shown that the combination of high |WSS| and high WSSG may result in the aneurysm rupture. We showed similar results; the combination of high |WSS| and high WSSD^+^ may result in the aneurysm rupture. It is worth to know the difference between WSSG and WSSD^+^. The former only represents the gradient of the magnitude of WSS but does not include WSS directions. The latter is in the same order of WSSG but can identify that, at the dangerous location, high |WSS| regions must stretch the aneurysm luminal surface. At low |WSS| regions, high OSI points are considered to be dangerous for aneurysm rupture, as WSS changes its directions at those points [29]. In this study, we further identity that if the directional change of WSS results in the alternation of a specific area between “tensile” (WSSD^+^) to “compressive” (WSSD^−^), it is more likely to be dangerous. In one word, WSSD based analysis further highlights the dangerous factors.

### 4.3. The Limitation of the Current Study

One of the limitations of this study is the small number of ruptured cases examined. We should highlight that the aneurysm cases were obtained from long-term patient follow-up observations conducted in the clinic. The ruptured aneurysms were strictly selected from images taken prior to rupture (not after rupture for most other studies). The selected patients were all of similar ages, all being female, nonsmokers with no family history of aneurysm rupture. The aneurysms were located at the same loci (ICA) and exhibited similar sizes. We are moreover keeping a record of all patient data and will continually introduce new cases into future research.

## 5. Conclusion

A new concept of WSSD was proposed in this paper, which considers both WSS magnitude and effective directions in the prediction of aneurysm rupture. Based on WSSD, two risk factors RFA and RFB are derived, which can be calculated through the use of CFD with a high-order scheme. Our results revealed that aneurysms with high values of RFA and RFB are under high risk of rupture. 

## References

[1] Teunissen LL, Rinkel GJE, Algra A, van Gijn J (1996). Risk factors for subarachnoid hemorrhage a systematic review. *Stroke*.

[2] Vlak MH, Rinkel GJE, Greebe P, Van Der Bom JG, Algra A (2011). Trigger factors and their attributable risk for rupture of intracranial aneurysms: a case-crossover study. *Stroke*.

[3] Rinkel GJE, Djibuti M, Algra A, Van Gijn J (1998). Prevalence and risk of rupture of intracranial aneurysms: a systematic review. *Stroke*.

[4] de Rooij NK, Linn FHH, van der Plas JA, Algra A, Rinkel GJE (2007). Incidence of subarachnoid haemorrhage: a systematic review with emphasis on region, age, gender and time trends. *Journal of Neurology, Neurosurgery and Psychiatry*.

[5] Phillips LH, Whisnant JP, O’Fallon WM, Sundt TM (1980). The unchanging pattern of subarachnoid hemorrhage in a community. *Neurology*.

[6] Kassell NF, Torner JC, Haley EC, Jane JA, Adams HP, Kongable GL (1990). The International cooperative study on the timing of aneurysm surgery. Part 1: overall management results. *Journal of Neurosurgery*.

[7] Wermer MJ, van der Schaaf IC, Algra A, Rinkel GJE (2007). Risk of rupture of unruptured intracranial aneurysms in relation to patient and aneurysm characteristics: an updated meta-analysis. *Stroke*.

[8] Baharoglu MI, Schirmer CM, Hoit DA, Gao B, Malek AM (2010). Aneurysm inflow-angle as a discriminant for rupture in sidewall cerebral aneurysms: morphometric and computational fluid dynamic analysis. *Stroke*.

[9] Xiang J, Natarajan SK, Tremmel M (2011). Hemodynamic-morphologic discriminants for intracranial aneurysm rupture. *Stroke*.

[10] Forget TR, Benitez R, Veznedaroglu E (2001). A review of size and location of ruptured intracranial aneurysms. *Neurosurgery*.

[11] Dhar S, Tremmel M, Mocco J (2008). Morphology parameters for intracranial aneurysm rupture risk assessment. *Neurosurgery*.

[12] Rahman M, Smietana J, Hauck E (2010). Size ratio correlates with intracranial aneurysm rupture status: a prospective study. *Stroke*.

[13] Shojima M, Oshima M, Takagi K (2004). Magnitude and role of wall shear stress on cerebral aneurysm: computational fluid dynamic study of 20 middle cerebral artery aneurysms. *Stroke*.

[14] Dolan JM, Kolega J, Meng H (2013). High wall shear stress and spatial gradients in vascular pathology: a review. *Annals of Biomedical Engineering*.

[15] Cebral JR, Castro MA, Burgess JE, Pergolizzi RS, Sheridan MJ, Putman CM (2005). Characterization of cerebral aneurysms for assessing risk of rupture by using patient-specific computational hemodynamics models. *American Journal of Neuroradiology*.

[16] Shojima M, Oshima M, Takagi K (2005). Role of the bloodstream impacting force and the local pressure elevation in the rupture of cerebral aneurysms. *Stroke*.

[17] Hassan T, Timofeev EV, Saito T (2005). A proposed parent vessel geometry-based categorization of saccular intracranial aneurysms: computational flow dynamics analysis of the risk factors for lesion rupture. *Journal of Neurosurgery*.

[18] Goubergrits L, Schaller J, Kertzscher U (2012). Statistical wall shear stress maps of ruptured and unruptured middle cerebral artery aneurysms. *Journal of the Royal Society Interface*.

[19] Meng H, Wang Z, Hoi Y (2007). Complex hemodynamics at the apex of an arterial bifurcation induces vascular remodeling resembling cerebral aneurysm initiation. *Stroke*.

[20] Traub O, Berk BC (1998). Laminar shear stress: mechanisms by which endothelial cells transduce an atheroprotective force. *Arteriosclerosis, Thrombosis, and Vascular Biology*.

[21] Qian Y, Takao H, Umezu M, Murayama Y (2011). Risk analysis of unruptured aneurysms using computational fluid dynamics technology: preliminary results. *American Journal of Neuroradiology*.

[22] Qian Y, Liu JL, Itatani K, Miyaji K, Umezu M (2010). Computational hemodynamic analysis in congenital heart disease: simulation of the Norwood procedure. *Annals of Biomedical Engineering*.

[23] Zhang Y, Furusawa T, Sia SF, Umezu M, Qian Y (2013). Proposition of an outflow boundary approach for carotid artery stenosis CFD simulation. *Computer Methods in Biomechanics and Biomedical Engineering*.

[24] Fisher AB, Chien S, Barakat AI, Nerem RM (2001). Endothelial cellular response to altered shear stress. *American Journal of Physiology*.

[25] Zeng Z, Chung BJ, Michael D, Robertson AM (2009). An in-vitro device for evaluation of cellular response to flows found at the apex of bifurcations. *Advances in Mathematical Fluid Mechanics*.

[26] Meng H, Metaxa E, Gao L (2011). Progressive aneurysm development following hemodynamic insult: laboratory investigation. *Journal of Neurosurgery*.

[27] Lu G, Huang L, Zhang XL (2011). Influence of hemodynamic factors on rupture of intracranial aneurysms: patient-specific 3D mirror aneurysms model computational fluid dynamics simulation. *American Journal of Neuroradiology*.

[28] Shimogonya Y, Ishikawa T, Imai Y, Matsuki N, Yamaguchi T (2009). Can temporal fluctuation in spatial wall shear stress gradient initiate a cerebral aneurysm? A proposed novel hemodynamic index, the gradient oscillatory number (GON). *Journal of Biomechanics*.

[29] Meng H, Tutino VM, Xiang J, Siddiqui A (2013). High WSS or low WSS? Complex interactions of hemodynamics with intracranial aneurysm initiation, growth, and rupture: toward a unifying hypothesis. *American Journal of Neuroradiology*.

